# Population Genetic Architecture of the *Streptococcus suis* Antigen HP0197

**DOI:** 10.3390/vetsci13040376

**Published:** 2026-04-13

**Authors:** Guopeng Mei, Junfeng Zhang, Lijun Guan, Shangbo Ning, Yun Xue, Zhanqin Zhao

**Affiliations:** 1 Luoyang Key Laboratory of Animal Bacterial Infectious Disease Prevention and Control Technology, College of Animal Science and Technology, Henan University of Science and Technology, Luoyang 471000, China; my1025229015@163.com (G.M.); jfzhang018@163.com (J.Z.); gljguanlijun@163.com (L.G.); sning@hasut.edu.cn (S.N.); xueyun6688@163.com (Y.X.); 2 Microbial Molecular Testing Technology Laboratory, College of Medical Technology and Engineering, Henan University of Science and Technology, Luoyang 471000, China

**Keywords:** *Streptococcus suis*, bioinformatics analysis, phylogroup, subunit vaccine

## Abstract

*S. suis* is a major zoonotic infectious disease whose serological diversity brings challenges to vaccine development. Based on the whole-genome data of 169 *S. suis* strains, this study conducted a systematic bioinformatics analysis of the surface antigen protein HP0197 that reveals its distribution characteristics, sequence diversity, domain composition and antigenic epitope distribution. The results showed that the HP0197 gene, which has a detection rate of 91.72%, can be divided into seven major phylogroups (I–VII) and the following two structural types: short form (HP0197-S) and long form (HP0197-L). All sequences contained signal peptides, transmembrane structures, LPXTG anchoring motifs, as well as conserved GAGBD and G5 domains, among which tandem repeats of the G5 domain existed in the long HP0197-L type. Tertiary structure prediction indicated that HP0197 has a spatial architecture of “conserved at both ends and flexible in the middle”, in which B-cell epitopes are mainly enriched near the GAGBD and G5 domains, suggesting these regions are the key targets for inducing cross-immune protection. It systematically elucidates the diversity and structural characteristics of the HP0197 protein from the perspective of population genetics, which provides a theoretical basis for optimizing existing subunit vaccines, designing broad-spectrum multi-epitope vaccines and exploring novel anti-infection strategies.

## Simple Summary

*Streptococcus suis* (*S. suis*) disease is a critical zoonotic infectious disease, and its serological diversity presents challenges to vaccine development. Based on the whole-genome data of 169 Streptococcus suis strains, this study performed a systematic bioinformatics analysis of the surface antigen protein HP0197. The results revealed that the detection rate of the HP0197 gene was 91.72%, and the amino acid sequences could be categorized into seven major phylogenetic groups (I–VII), with the following two structural types: the short type (HP0197-S) and the long type (HP0197-L). All sequences contained signal peptides, transmembrane structures, LPXTG anchoring motifs, and conserved GAGBD and G5 domains; notably, tandem repeats of the G5 domain were observed in the long-type HP0197-L. Tertiary structure prediction indicated that HP0197 exhibits a spatial architecture characterized by “conserved termini and a flexible central region”. B-cell epitopes were primarily enriched in the vicinity of the GAGBD and G5 domains, suggesting these regions are key targets for inducing cross-immune protection. From the perspective of population genetics, this study systematically elucidates the diversity pattern and structural features of the HP0197 protein, providing a theoretical foundation for optimizing existing subunit vaccines, designing broad-spectrum multi-epitope vaccines, and exploring novel anti-infection strategies.

## 1. Introduction

*S. suis* is a major zoonotic pathogen which not only represents an important bacterial pathogen for the global swine industry, but also poses a severe threat to public health security [1,2]. It can breach host defense barriers and cause invasive multisystemic infections including sepsis, meningitis, arthritis and endocarditis [3]. Its pathogenesis involves multiple processes such as adhesion and colonization, immune evasion and transbarrier invasion. Moreover, its virulence varies significantly among strains of different serotypes, which poses great challenges to clinical prevention and control. Currently, 29 serotypes and a variety of novel capsular locus variants have been identified based on differences in capsular polysaccharide antigens [4,5,6,7,8,9,10,11]. The lack of effective cross-protection among these serotypes accounts for the unstable and inconsistent efficacy of conventional inactivated vaccines. In this context, vaccination, as a core strategy for the control of swine streptococcosis, is urgently required to develop toward higher safety and broad-spectrum protection. Subunit vaccines have gradually emerged as a major focus for novel vaccine development due to their advantages including well-defined antigen components, high safety and ease of large-scale production. Screening conserved antigens capable of inducing broad cross-protective immunity represents a pivotal scientific issue that determines the success of vaccine development.

HP0197 was identified from cell wall-associated proteins of *S. suis* serotype 2 by Zhang Anding et al. using an immunoproteomic approach. Studies have confirmed that it contains a typical LPXTG motif, is localized on the bacterial surface, and can stimulate the host to produce an immune response [12,13,14]. Yan Xiaojie et al. analyzed the typical structural characteristics of its C-terminal G5 domain and speculated that this domain may be associated with cell adhesion. HP0197 can interact with host Hep-2 cells, and this interaction can be specifically inhibited by heparin, suggesting that glycosaminoglycans on the host cell surface may serve as its receptor. Further studies confirmed that HP0197 binds to cell surface glycosaminoglycans via a novel 18 kDa α-helix-rich N-terminal domain (GAGBD). The key lysine binding sites were identified, clarifying the molecular mechanism by which HP0197 acts as a bacterial adhesin to mediate host cell attachment [15]. Furthermore, HP0197 is involved in the global carbon metabolism and virulence regulatory network of the bacterium. It affects the phosphorylation level of HPr, a cofactor of the global transcriptional regulator CcpA, thereby regulating CcpA activity and ultimately affecting capsular polysaccharide synthesis and overall virulence [16].

Subsequent studies showed that immunization of mice and piglets with recombinant HP0197 protein can induce a significant humoral immune response and provide effective protection against lethal challenge with SS2 [13]. In 2019, a bivalent subunit vaccine against *S. suis* and *Haemophilus parasuis* was licensed for commercial production, which consists of *S. suis* proteins HP0197 and HP1036, as well as *H. parasuis* proteins 06257 and PalA. Although HP0197 has been confirmed to be widely distributed and highly conserved in *S. suis*, systematic genome-wide analyses of its sequence polymorphism, phylogenetic relationships among different strains, and the effects of these variations on its structure and function remain limited. In this study, we conducted phylogenetic analysis, domain composition comparison, and epitope prediction of the HP0197 protein using 169 complete genomes of Streptococcus suis available in the GenBank database (https://www.ncbi.nlm.nih.gov/genome/, accessed on 5 January 2026). Although HP0197 has been recognized as a promising vaccine candidate, its genetic diversity, structural conservation, and immunodominant regions across globally distributed strains remain poorly characterized. To address this knowledge gap, we systematically evaluated the HP0197 family to elucidate its genetic diversity, structural conservation, and immunodominant regions, thereby providing a theoretical basis for optimizing current vaccines and guiding the development of future broad-spectrum vaccine strategies.

## 2. Materials and Methods

### 2.1. Distribution, Amino Acid Sequence and Phylogenetic Analysis of HP0197

To investigate the distribution characteristics and evolutionary relationships of HP0197 in *S. suis*, the keyword “*Streptococcus suis*” was searched in the GenBank database. After removing duplicate strains, low-quality assemblies, and closely related isolates, a total of 169 complete whole-genome sequences of *S. suis* were obtained (statistics were limited to strains with complete sequences, with a deadline of 5 January 2026). Using the HP0197 gene of *S. suis* strain SC19 as the seed sequence, the nucleotide sequence of HP0197 from strain SC19 was aligned with the whole-genome database of the 169 registered *S. suis* strains (E-value threshold set to 1 × 10^−5^, and other parameters kept unchanged) via NCBI BLASTn (version 2.17.0; National Center for Biotechnology Information, Bethesda, MD, USA) (https://blast.ncbi.nlm.nih.gov/Blast.cgi, accessed on 5 January 2026). A total of 155 *S. suis* strains carrying the HP0197 sequence were screened out, and the presence, coverage (the percentage of the aligned region in the full length of the target sequence) and sequence similarity of HP0197 in each strain were statistically analyzed. Using MEGA 12.0 software, the Subtree-Pruning-Regrafting (SPR) model was selected as the substitution model, ClustalW was used as the alignment method, and Partial Deletion was set as the gap treatment strategy. The maximum-likelihood phylogenetic tree of 155 HP0197 amino acid sequences was constructed, and branch confidence was evaluated by Bootstrap with 1000 replicates. The phylogenetic tree was visually modified using the online tool iTOL (https://itol.embl.de, accessed on 6 January 2026).

### 2.2. Preliminary Analysis and Classification of HP0197 Sequence Variation Based on Multiple Sequence Alignment

To further analyze the sequence diversity of HP0197, the HP0197 nucleotide sequence from the complete genome of *S. suis* strain SC19 was used as the reference, and the HP0197 amino acid sequences and their accession numbers of 155 *S. suis* strains were collected. The accession numbers were submitted in batch to the online tool COBALT (https://www.ncbi.nlm.nih.gov/tools/cobalt/cobalt.cgi#i, accessed on 6 January 2026) for multiple sequence alignment. A total of 13 abnormal sequences were removed, including those with too few amino acids or lacking highly conserved domains, which could help ensure data accuracy. Sequences with abnormal lengths were removed to ensure data quality. Sequence variation characteristics were analyzed through alignment maps, and seven types of amino acid sequences with typical differences were screened out. Insertion/deletion sites, conserved motifs and hypervariable region distribution in each phylogroup were systematically analyzed to clarify their differential characteristics and stable structural regions, laying a foundation for subsequent domain prediction and antigenic epitope analysis.

### 2.3. Prediction of Conserved Domains in Different Phylogroups of HP0197

To clarify the structural characteristics of each phylogroup of HP0197, representative amino acid sequences were selected as the sequence with the highest frequency (i.e., the most frequently observed HP0197 sequence) within each phylogroup. These representative sequences were submitted to the CD-Search and InterPro databases for conserved domain prediction, with the parameter set to “Standard” or “Summary” and the remaining parameters set to default. Domains predicted by both tools were defined as the conserved domains of each phylogroup, and the names and positions of each domain were recorded. The online software IBS 2.0 (https://ibs.renlab.org/#/home, accessed on 7 January 2026) was used for visual drawing and optimization of the conserved domains. Differences and similarities in the structural composition of different phylogroups were revealed by comparative analysis [17].

### 2.4. General Bioinformatics Analysis of Different Phylogroups of HP0197

To comprehensively evaluate the molecular characteristics of each phylogroup of HP0197, one representative amino acid sequence was selected from each of phylogroups I–VI. A series of bioinformatics tools were used to analyze and predict the properties of the HP0197 protein. ProtParam and ProtScale were used to predict the physicochemical properties and hydrophilicity–hydrophobicity of each representative sequence; SignalP-6.0, TMHMM-2.0 and PSORTB were used to predict signal peptides, transmembrane structures and subcellular localization, respectively; SOPMA and AlphaFold3 were used to predict the secondary and tertiary structures, respectively. The tertiary structure was optimized and displayed using the online software PyMOL version 3.1.6.1 (Schrödinger, Inc., New York, NY, USA) (Table 1).

### 2.5. Prediction and Analysis of B-Cell Epitopes in Different Phylogroups of HP0197

To evaluate the immunogenic potential of HP0197 in each phylogroup, the representative sequences that were selected from phylogroups I–VI were submitted to the online tools ABCpred (threshold: 0.85, window size: 16; however, an excessively low threshold could reduce the accuracy of predicted B-cell epitopes) and BepiPred-3.0 (default parameters), which are widely used for B-cell epitope prediction. The consensus epitopes that were predicted by both tools were regarded as credible epitopes. Shared and phylogroup-specific epitopes that were identified through comparative analysis were visualized with IBS 2.0 software.

## 3. Results

### 3.1. Distribution of HP0197 in S. suis

A total of 169 complete whole-genome sequences of *S. suis* that were retrieved from the GenBank database were analyzed in this study. BLASTn alignment was performed using the “Megablast” program against the HP0197 nucleotide sequence of *S. suis* strain. SC19 showed that 155 strains harbored the HP0197 gene, with a positive rate of 91.7% and sequence similarity ranging from 90% to 100%. The HP0197 gene exists only in *S. suis*, and no homologous genes exist in other *Streptococcus* species such as *S. equi* subsp. zooepidemicus, *S. pneumoniae*, and *S. pyogenes* or in any other organisms that have been deposited in GenBank. On the basis of DNA fragment length, the HP0197 gene that was identified in the 155 strains was roughly classified into the following six groups: 1686 bp (27 strains), 1587 bp (nine strains), 1560 bp (12 strains), 2409 bp (19 strains), 2511 bp (31 strains), 2109 bp (six strains), together with 51 strains that displayed irregular lengths. BLAST analysis that employed the HP0197 sequence of *S. suis* strain SC19 as the reference showed that 45 strains had a coverage of 90–100% with similarity ≥ 96.40%, 94 strains had 46–59%, 12 strains had 60–67%, three strains had 76–87%, and one strain had a coverage of 43%. A second BLAST analysis that was conducted for the 94 strains with the HP0197 sequence of *S. suis* strain NJ3 as the reference revealed that 41 strains had a coverage of 90–100%, 31 strains had 80–82%, and 22 strains had 26–31%. These results indicated that HP0197 may have at least seven distinct sequence types. Among the 155 *S. suis* strains that carried the HP0197 gene, 115 were isolated from swine, 33 from human, one from bovine, one from Trichogaster sp., and five whose host origin could not be determined. Of these strains, 57 were from China, 41 from Thailand, 11 from Canada, 10 from the USA, 10 from the Netherlands, 25 from other countries, and one whose geographic origin was unknown. Serotype information that was available for 142 strains was obtained from GenBank annotations and the PubMed literature: serotype 1 (six), serotype 2 (50), serotype 3 (eight), serotype 4 (eight), serotype 5 (10), serotype 9 (nine), serotype 31 (19), other serotypes (19), and 13 non-typeable strains (Supplementary Table S1).

### 3.2. Phylogenetic Analysis and Genotyping of HP0197 Amino Acid Sequences

**Figure 1:**
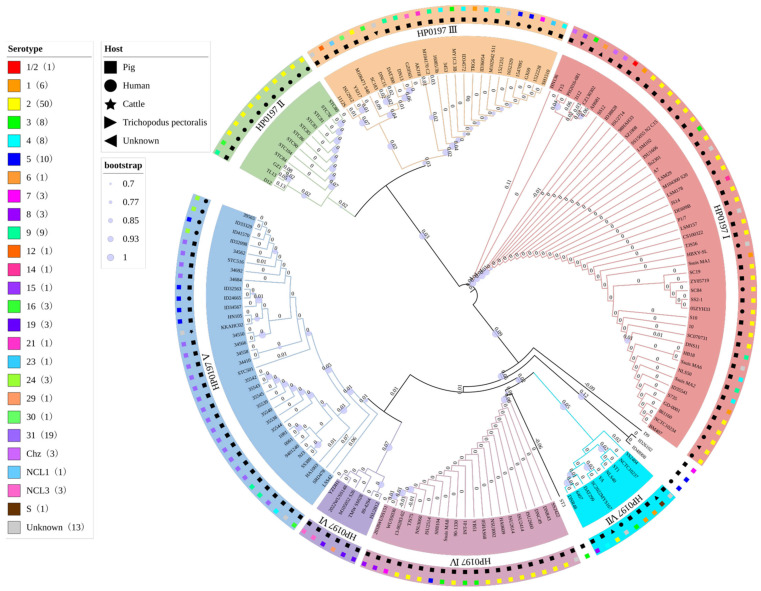
Phylogenetic tree of HP0197 from 155 *S. suis* strains.

**Figure 2:**
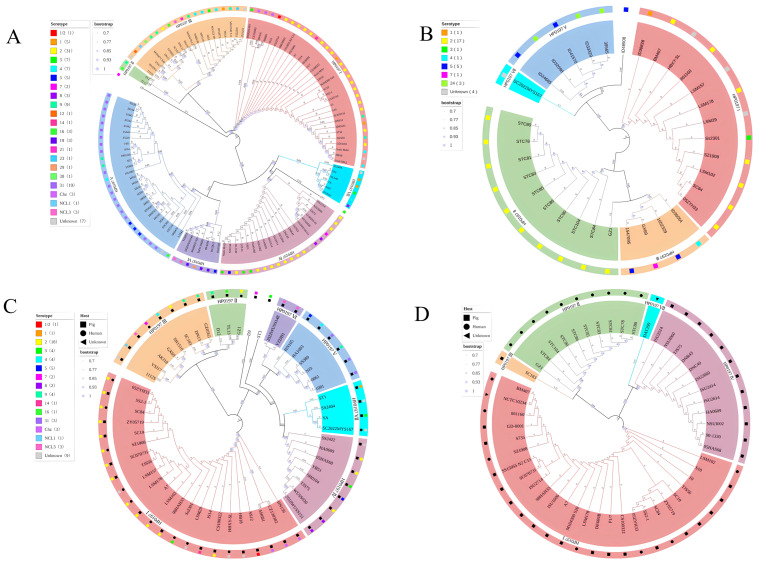
Phylogenetic analysis of HP0197 in *S. suis* from different sources, including porcine strains (**A**), human strains (**B**), Chinese strains (**C**), and serotype 2 strains (**D**).

Phylogenetic analysis indicated that the HP0197 amino acid sequences from 155 *S. suis* strains could be divided into seven phylogroups I–VII and four ungrouped sequences, which accounted for 2.28% of all isolates (Figure 1; Table 2). Phylogroups I–VI corresponded to the six categories classified by DNA fragment length in Section 2.1, while phylogroup VII and the four ungrouped sequences matched those with irregular length profiles. A total of 115 swine-derived strains carried HP0197 sequences covering all seven phylogroups: 32 (27.83%), two (1.74%), 19 (16.52%), 20 (17.39%), 27 (23.48%), six (5.22%), and six (5.22%) respectively, together with three ungrouped strains (2.61%). The 33 human-derived strains carried HP0197 sequences from five phylogroups I, II, III, V and VII, with no sequences from phylogroups IV or VI. This result showed that strains carrying these five HP0197 types lacked host specificity and could transmit between pigs and humans (Figure 2; Table 3). All 20 sequences in phylogroup IV were found only in swine-derived strains, suggesting that strains with phylogroup IV HP0197 were host-specific and unlikely to infect humans. Phylogroup VI showed a similar pattern. Based on the current sample size, we hypothesize that strains harboring phylogroup IV HP0197 may be host-specific and unlikely to infect humans. Phylogroup VI showed a similar pattern, but further verification is needed due to the small sample size of only six strains. Notably, nine of the 12 strains with phylogroup II HP0197 were isolated from human patients infected by *S. suis* serotype 2 during an outbreak in Thailand in 2021 caused by eating raw pork, and all these strains belonged to the novel ST1656 clonal complex [18]. The distribution of HP0197 phylogroups among different serotypes (Table 2) demonstrated that 50 serotype 2 strains contained sequences from phylogroups I–IV and VII, but not from phylogroups V or VI. Based on this observation, we speculate that these two phylogroups may be associated with *S. suis* serotypes. The relationship between these phylogroups and the pathogenicity of related serotypes deserves further investigation. In addition, all 19 serotype 31 strains carried only phylogroup V HP0197. Among them, 16 strains came from pig herds in different regions of Thailand covering 11 distinct STs, and three strains were derived from pig herds in different regions of China [19]. This supported a close correlation between phylogroup V and *S. suis* serotype 31. Meanwhile, phylogroup V HP0197 was not detected in any serotype 2 strain. These results suggested that HP0197 from different phylogroups may exert a significant impact on the infection and immune mechanisms of host *S. suis*. Further studies on the underlying mechanisms may contribute to understanding the cross-immune protective efficacy of HP0197 from different phylogroups.

**Table 2:** Phylogenetic analysis of HP0197 (serotype).

Serotype	I	II	III	IV	V	VI	VII	Other	Total
1/2	1	- ^1^	-	-	-	-	-	-	1
1	3	-	1	-	-	-	2	-	6
2	26	10	1	12	-	-	1	-	50
3	2	-	1	2	-	-	1	1	8
4	2	-	4	-	1	-	1	-	8
5	-	-	2	1	5	-	-	2	10
6	1	-	-	-	-	-	-	-	1
7	-	-	1	1	-	-	-	1	3
8	-	-	-	3	-	-	1	-	3
9	2	1	4	-	2	-	-	-	9
12	-	-	1	-	-	-	-	-	1
14	1	-	-	-	-	-	-	-	1
15	1	-	-	-	-	-	-	-	1
16	-	1	1	-	1	-	-	-	3
19	-	-	-	-	-	3	-	-	3
21	-	-	1	-	-	-	-	-	1
23	-	-	1	-	-	-	-	-	1
24	-	-	-	-	3	-	-	-	3
29	-	-	-	-	-	1	-	-	1
30	-	-	1	-	-	-	-	-	1
31	-	-	-	-	19	-	-	-	19
Chz	3	-	-	-	-	-	-	-	3
NCL1	-	-	1	-	-	-	-	-	1
NCL3	-	-	1	-	-	2	-	-	3
S	-	-	-	-	-	-	1	-	1
Unknow	5	-	3	1	2	-	2	-	13
Total	47	12	24	20	33	6	9	4	155
Percent (%)	30.32	7.74	15.48	13.55	21.29	3.87	5.81	1.94	100

^1^ no corresponding strain.

**Table 3:** Phylogenetic analysis of HP0197 (host).

Host	I	II	III	IV	V	VI	VII	Other	Total
Pig	32	2	19	20	27	6	6	3	115
Human	12	10	4	-	5	-	1	1	33
Cattle	- ^1^	-	-	-	1	-	-	-	1
Trichopodus pectoralis	1	-	-	-	-	-	-	-	1
Unknow	2	-	1	-	-	-	2	-	5
Total	47	12	24	20	33	6	9	4	155

^1^ no corresponding strain.

### 3.3. Multiple Sequence Alignments and Conserved Domain Predictions of HP0197 in Different Phylogroups

**Figure 3:**
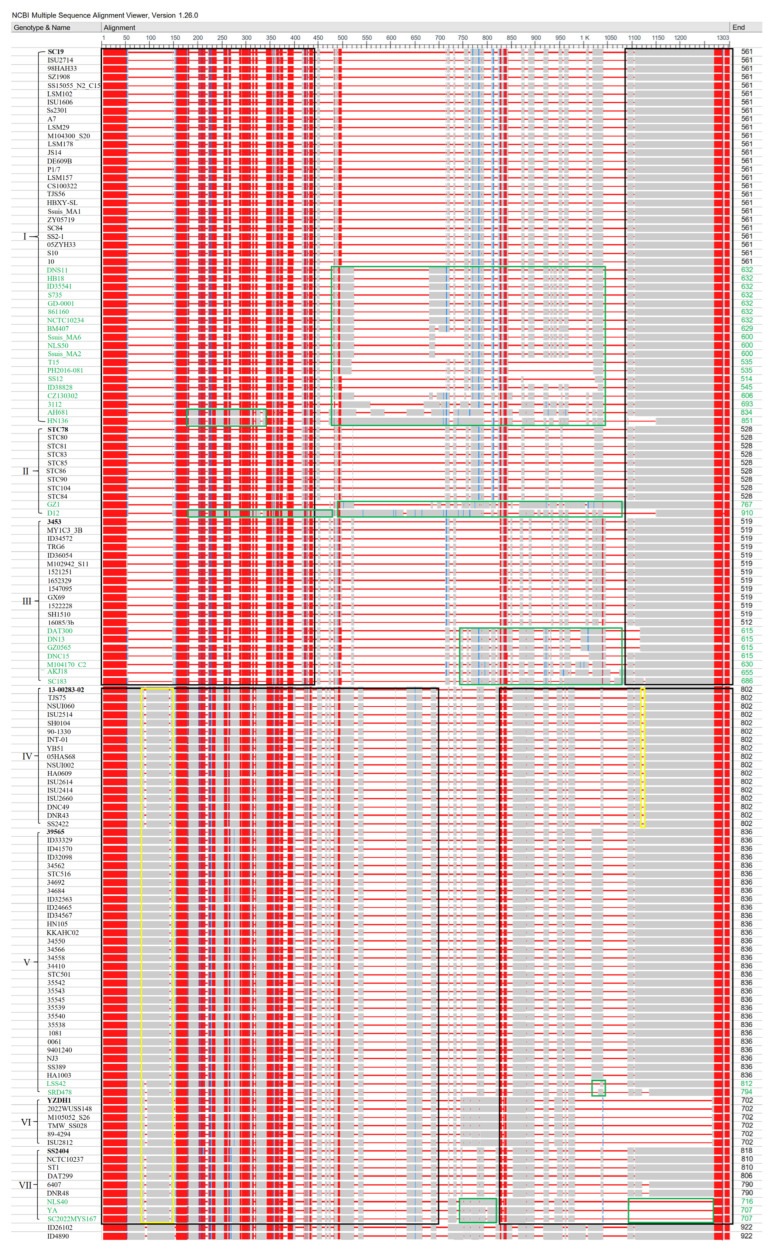
Amino acid alignment of HP0197 protein. Green regions represent sequences with variations within each phylogroup; black regions represent highly conserved sequences in both short-type and long-type HP0197; yellow regions represent variable sequences within the highly conserved regions.

Based on the phylogenetic tree classification that divided all sequences into seven phylogroups I–VII, multiple sequence alignment of HP0197 amino acid sequences from 155 *S. suis* strains was performed using COBALT. The results (Figure 3) showed that the N-terminal signal peptide (1–39 aa) and the C-terminal LPXTG anchor motif were highly conserved in all HP0197 sequences. According to the length differences in the full-length alignment (1–1303 aa), HP0197 was classified into two types. Among the 76 sequences from phylogroups I–III, 73 sequences except those from strains AH681, HN136 and D12 ranged from 512 to 686 aa, which were defined as the short-type HP0197 and designated HP0197-S. The 64 sequences from phylogroups IV–VII ranged from 702 to 836 aa, which were defined as the long-type HP0197 and designated HP0197-L.

HP0197-S sequences were highly homologous in regions 1–440 aa and 1090–1303 aa, and variations were mainly concentrated in the central Loop region (441–1089 aa) (Figure 3) [15]. Each phylogroup contained highly homologous sequences, including 26 sequences (561 aa) in phylogroup I, nine sequences (528 aa) in phylogroup II, and 13 sequences (519 aa) in phylogroup III. The variations in the remaining sequences were located in different parts of the Loop region as follows: 441–1020 aa in phylogroup I, 500–1089 aa in phylogroup II, and 850–1089 aa in phylogroup III. Based on the available data, we speculate that the divergence among the 73 HP0197-S sequences may be mainly concentrated in the central Loop region (441–1089 aa).

HP0197-L sequences exhibited fragment variations in the central Loop region 750–850 aa and the C-terminal region 1120–1140 aa, as well as individual site differences at N-terminal regions 90–95 aa and 145–150 aa (Figure 3). In addition, all sequences in phylogroup VI and three sequences in phylogroup VII showed a deletion in the C-terminal region 1090–1270 aa, which shared high homology with the segment 1170–1270 aa, suggesting that the deletion may be associated with internal domain duplication or recombination. Among these HP0197-L sequences, all 17 sequences in phylogroup IV and all six sequences in phylogroup VI were highly consistent. Among the 32 sequences in phylogroup V, 30 were highly homologous with a length of 836 aa, and the other two sequences showed variations at 1020–1040 aa. The nine sequences in phylogroup VII were divided into two groups as follows: six sequences with lengths ranging from 790 to 818 aa, and three sequences with a deletion in the C-terminal region 1090–1270 aa.

In conclusion, among the 155 *S. suis* strains carrying HP0197, the majority of sequences except 18 out of 155 can be generally classified into short-type and long-type. These sequences were highly conserved only in the N-terminal signal peptide region and the C-terminal region (1090–1303 aa), and several small fragments at the N-terminus also showed high similarity. The majority of HP0197-S sequences (48/73) were highly conserved at N-terminal 1–440 aa and C-terminal 1090–1303 aa, with variations mainly located in the central Loop region (441–1089 aa). The majority of HP0197-L sequences (55/64) shared conserved regions at N-terminal 1–700 aa and C-terminal 1090–1303 aa. This indicates that there are substantial differences between the short-type and long-type sequences.

**Figure 4:**
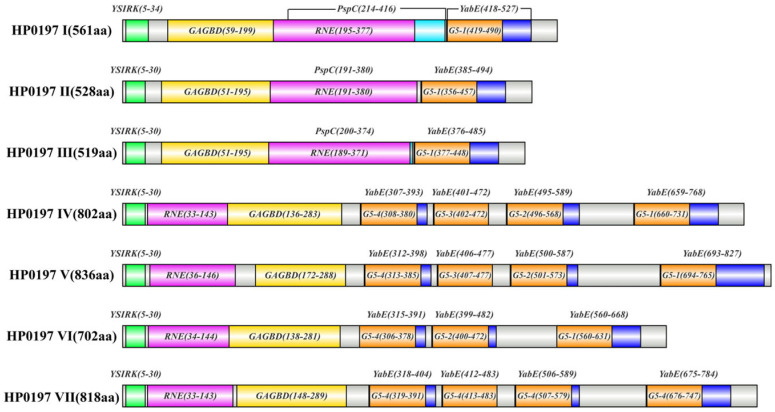
Conserved domain prediction of HP0197 in different phylogenetic phylogroups.

Based on the structural analysis results that were obtained from CD-Search and InterPro, HP0197 can be classified into two structural types, which are consistent with the short-type HP0197-S and long-type HP0197-L that were classified by COBALT. The first structural type (HP0197-S; phylogroups I–III), which corresponds to the short form, mainly includes the YSIRK signal peptide, GAGBD that can bind glycosaminoglycans (GAGs) on host cell surfaces, RNE (ribonuclease E) that is located in the Loop region, YabE that is an uncharacterized conserved protein, and the G5 domain that mainly binds N-acetylglucosamine [15,20,21,22]. The second structural type, which corresponds to the aforementioned HP0197-L (phylogroups IV–VII), mainly includes the YSIRK signal peptide, GAGBD, RNE, and four YabE domains in phylogroups IV, V, and VII, whereas phylogroup VI only contains three YabE domains and the G5 domain. In addition, non-domain insertion fragments exist in phylogroups IV–VII (Figure 4). The domain distribution pattern of phylogroup VII, which is highly similar to that of phylogroup IV, is consistent with the phylogenetic relationships that are shown in the phylogenetic tree. Given the high similarity in structural composition that exists between phylogroup VII and phylogroup IV, the subsequent analysis of physicochemical properties and antigenic epitopes of HP0197-L will focus on phylogroups IV, V, and VI, which means no separate discussion will be made for phylogroup VII.

BLAST analysis showed that the coverage of GAGBDs among groups I–VII ranged from 82% to 100%, while their sequence similarity was only 41.13–45.39%, which implies that the domain maintains its overall framework with certain sequence variations. The RNE domain, which is distributed in different groups, exhibits great sequence divergence, and the amino acid sequences of different G5 domains also show mere differences. G5-1 is highly conserved across the seven groups, and G5-2 is highly similar to G5-3. However, the similarity between G5-1 and the other three G5 domains was only 32.39–47.89%. In addition, hydrophobicity analysis of the G5 domains indicated that hydrophobic amino acids in G5-1 account for a significantly higher proportion, which results in an alteration of its structural features. The other three G5 1-4 domains share similar hydrophobic amino acid contents and present a distinct “two valleys and one peak” structure, suggesting that these three domains may be tandem repeat units. Taken together, based on the above results, we speculate that the enhanced hydrophobicity of G5-1 may participate in regulating the interactions between *S. suis* and the external environment, which still needs to be further verified by experiments including molecular docking.

### 3.4. General Bioinformatic Analysis of HP0197 in Different Phylogroups

Analysis of the physicochemical properties of HP0197 proteins from phylogroups I–VI showed that all of them were acidic and hydrophilic proteins with medium to large molecular weights, which had theoretical isoelectric points (pI) ranging from 4.81 to 5.27 and grand average of hydropathicity (GRAVY) values from −0.715 to −0.858. All proteins contained the “LPXTG” cell wall anchor motif that is characteristic of Gram-positive bacterial surface proteins (Table 4), which suggests a possible localization to the cell wall and involvement in bacterial adhesion to the host.

**Figure 5:**
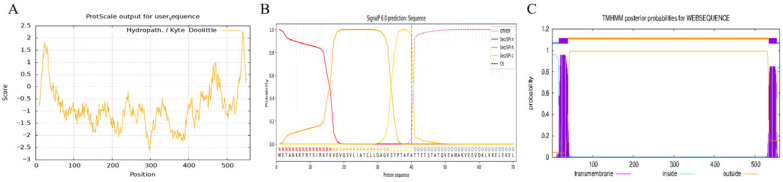
Hydrophobicity or hydrophilicity analysis, signal peptide prediction, and transmembrane domain prediction of HP0197 phylogroup I. Hydrophobicityor hydrophilicity analysis (**A**), signal peptide prediction (**B**), transmembrane domain prediction (**C**).

Prediction results of signal peptides, hydrophobicity and transmembrane domains consistently indicated that the signal peptide cleavage site of all HP0197 proteins was located at amino acids 40–41. In addition, there was a hydrophobic region at both the N-terminus and the C-terminus, among which the N-terminal hydrophobic region was highly consistent with the predicted signal peptide region, and the C-terminal hydrophobic region was highly consistent with the LPXTG cell wall anchor motif. Transmembrane domain prediction showed that the N-terminal region may also contain two transmembrane helices that are located within the signal peptide and near the N-terminus, respectively (Figure 5). These structural features, which were highly conserved during evolution, imply that the N- and C-termini of HP0197 jointly form the core structural framework of the protein that may synergistically participate in cell wall anchoring and surface display.

**Figure 6:**
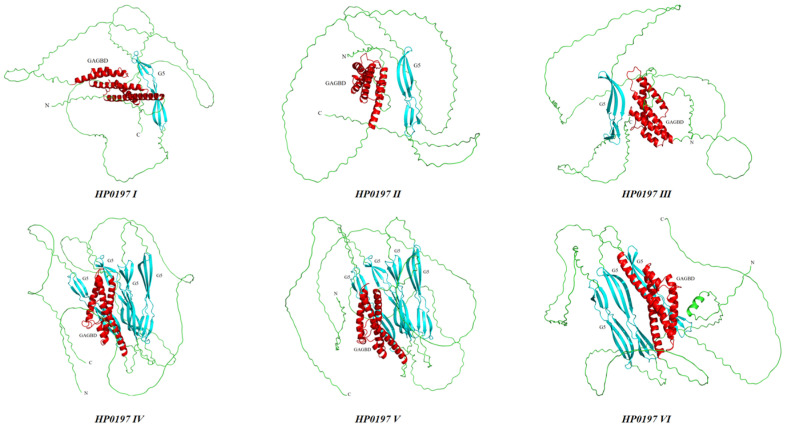
Predicted tertiary structure models of HP0197 proteins from six different phylogenetic phylogroups.

Secondary structure prediction revealed that HP0197 proteins were mainly composed of random coil (72.79–88.16%), which contained a small amount of α-helix (9.69–21.51%) and rare or absent β-sheet (Table 5). The tertiary structures of these six HP0197 sequences exhibit high pLDDT scores (90.3–97.8%) in the N-terminal α-helical region and C-terminal β-sheet region, while the pLDDT scores in other random coil regions are low, ranging from 51.2 to 55.3%. Tertiary structure prediction further uncovered a spatial architecture that is “conserved at both termini and flexible in the central region”: the N-terminus contained multiple α-helices, and the C-terminus formed a stable domain that consists of two three-stranded β-sheets (Figure 6), which was highly consistent with the multiple sequence alignment results. These structural features suggest that HP0197 may be anchored to the cell wall via the conserved C-terminal region, while the central flexible region may be involved in recognizing different host receptors that promote cross-host transmission.

This structural pattern, which is “conserved at both ends and flexible in the middle”, provides a structural basis for the phylogenetic diversity of HP0197. As shown in the previous multiple sequence alignment (Figure 3), sequence variations among different phylogroups were mainly concentrated in the central Loop region (441–1089 aa), whereas the functional domains at both termini such as GAGBD and G5 domains were highly conserved. The central region, which is mainly composed of random coil in the secondary structure without rigid folding constraints, exhibits high sequence plasticity that makes it more prone to mutation, insertion, deletion or even domain duplication (e.g., multiple G5 domains in phylogroups IV–VI). Such structural flexibility allows HP0197 to evolve diverse variants while maintaining core functions, which finally results in the phylogroups that were observed in this study.

### 3.5. Prediction of B-Cell Epitopes of HP0197 in Different Phylogroups

**Figure 7:**
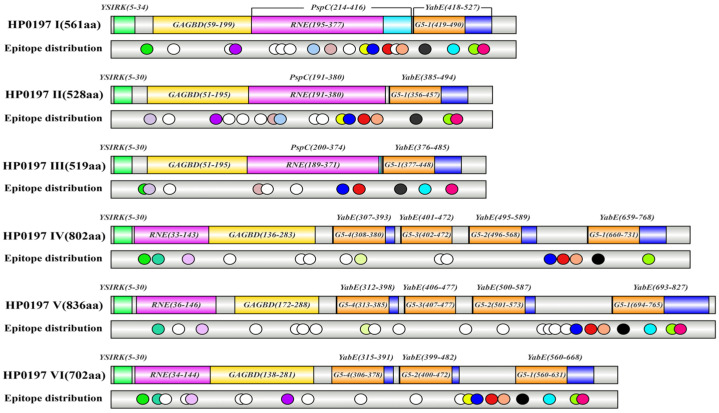
Predicted B-cell epitope sites of HP0197 proteins from six different phylogenetic phylogroups. Identical colors indicate shared epitopes; white indicates specific epitopes.

To evaluate the immunogenic potential of HP0197 proteins, one representative sequence from each of phylogroups I–VI was selected for B-cell epitope prediction. The results showed that multiple shared and specific epitopes existed among different phylogroups, whose distribution was closely related to the structural types of the protein (Figure 7; Table 6).

In HP0197-S type (phylogroups I–III), shared epitopes were mainly concentrated near the N-terminal GAGBD and the C-terminal G5 domain. For example, the epitope TETSTATQVEAMAKV (41–56) near the GAGBD was highly conserved in phylogroups I–III. The epitope ALPIKEETRYTASLPL (427–442, phylogroup I) near the G5 domain and its homologous sequences ALPIKEIERYDASLPL (394–409, phylogroup II) and ALPIKEIERYDASLAL (385–400, phylogroup III) also showed high similarity in HP0197-S.

In HP0197-L type (phylogroups IV–VI), shared epitopes were also enriched near the GAGBD and G5 domains. For instance, the epitope EPTVQITGTVSTENTN (60–75) in the GAGBD was present in phylogroups IV, V, and VI. The epitope KAEDSAPKTAVPEVAP (633–648, phylogroup IV; 667–682, phylogroup V; and 533–548, phylogroup VI) near the G5 domain and its homologous sequences were widely distributed in all long-type phylogroups.

HP0197-S also contains several core epitopes shared with HP0197-L, such as DKFKTDEVESDKQMPE (351–366, phylogroup I) and its homologous sequences that exist in phylogroups II–VI, and PKAEDMPKEEMPKSEQ (375–390, phylogroup I), which is highly conserved across all phylogroups. These epitopes were predicted in phylogroups I–VI, indicating that highly conserved antigenic regions also exist in the Loop region. In addition, with tandem duplication of G5 domains, HP0197-L exhibits new specific epitopes in the duplicated regions, such as EAPKTDKVESDKQMPE (626–641) and PSVQSNPTQSHKGAPS (778–793) in the second G5 domain of phylogroup V, and DKPKTDKVESDKQMPE (492–507) in the third G5 domain of phylogroup VI, which are absent in HP0197-S.

In conclusion, shared epitopes were mainly located at or near the N-terminal GAGBD, the C-terminal G5-1 domain, and specific conserved fragments in the Loop region. These regions were highly conserved during evolution, which suggests that they may be the core immunodominant epitopes of HP0197. In contrast, polymorphic epitopes were mainly distributed in the central hypervariable region (the Loop region excluding the conserved fragments mentioned above) and the duplicated G5 domains (G5-2, G5-3, and G5-4) of HP0197-L, which showed obvious type specificity. Because these hypervariable regions differed significantly among different phylogroups, and animal immune protection experiments indicated that most antigenic epitopes in the Loop region showed low protective efficacy (unpublished data), subunit vaccines targeting these regions may fail to induce broad cross-immune protection. Therefore, in the design of HP0197-related subunit vaccines, priority should be given to shared epitopes located in conserved domains (such as GAGBD and G5-1 domains), or multi-epitope vaccines covering multiple phylogroups should be designed. It should be noted that predictions based solely on linear epitopes have certain limitations, such as the inability to reflect conformational epitopes, surface accessibility, or the structural context of antigen–antibody interactions. Thus, the current analysis provides only a preliminary estimation, and further experimental validation of protective efficacy is required.

## 4. Discussion

*S. suis*, an important zoonotic pathogen, poses persistent challenges to vaccine development due to its high serotype diversity and antigenic variation. In this study, a systematic bioinformatic analysis of the *S. suis* HP0197 protein family was performed. For the first time, genome-wide phylogenetic classification, evolutionary relationships, sequence polymorphisms, and structural and antigenic characteristics of HP0197 were investigated. Based on 169 published whole-genome sequences, we revealed that HP0197 was widely distributed in *S. suis* (detection rate 91.72%) and highly homologous (>90%), while its amino acid sequences exhibited remarkable polymorphism and could be divided into seven major phylogroups (I–VII). This classification overcomes the previous fragmented understanding of HP0197 limited to specific serotypes or strains, and for the first time analyzes the overall evolutionary pattern of this antigen family at the population level. It lays an important foundation for studies on population genetics, structural diversity, and immunogenicity of the HP0197 protein in *S. suis*, and also provides an important theoretical basis for the optimization of existing subunit vaccines and the design of multi-epitope vaccines.

At the structural level, all HP0197 proteins contain hallmark elements of Gram-positive bacterial surface proteins: the YSIRK signal peptide, the LPXTG cell wall anchor motif, as well as functional domains including GAGBD and G5. Tandem duplication of the G5 domain was observed in the long-type HP0197-L (phylogroups IV–VII), implying potential functional differentiation or enhancement. Such duplication events may have arisen from replication slippage or unequal crossing over, contrasting with the deletions observed in the central Loop region of the short-type HP0197-S, which might reflect distinct evolutionary trajectories shaped by different host immune pressures or ecological niches. Tertiary structure prediction further revealed a spatial architecture of “conserved termini and flexible central region”, which provides a structural basis for sequence variation and explains why the central Loop region is the most polymorphic region. B-cell epitope prediction showed that immunodominant epitopes were mainly concentrated near the GAGBD and G5 domains and highly conserved among different phylogroups, whereas epitopes in the Loop region and duplicated G5 domains showed obvious type specificity. These findings identify key target regions for subsequent vaccine design.

On the basis of phylogenetic classification and analysis of its structure and diversity, this study further proposes an important inference: certain cross-protective efficacy may exist within the short-type HP0197-S (phylogroups I–III, 83/155), and similar cross-protection may also exist within the long-type HP0197-L (phylogroups IV–VII, 68/155). These two structural types may have originated from distinct evolutionary events—tandem domain duplication in HP0197-L versus central region deletion in HP0197-S—which could account for their divergent sequence features and potentially limited cross-immunity between them. However, due to significant differences in sequence and structure, effective cross-immune protection is likely absent between the two major types HP0197-S and HP0197-L. This inference raises an important warning about the application scope of current subunit vaccines developed based on a single HP0197 antigen and also explains why the protective efficacy of existing vaccines varies against different serotypes or strains. Nevertheless, this hypothesis still requires rigorous verification through systematic serological assays and animal immune protection experiments to clarify cross-immune reactivity among phylogroups and provide experimental evidence for vaccine design and application.

Although this study has depicted a relatively complete landscape of HP0197 diversity at the genomic level, many issues remain to be addressed from theoretical understanding to practical application. To address the limitations of the current dataset, future studies should expand the sample scope to include strains from more regions, hosts and serotypes, further improving the phylogenetic map of HP0197 and exploring potential associations between its distribution and strain pathogenicity or epidemiology. For the tandem duplication of the G5 domain, structural biology and immunological approaches should be combined to further dissect its functional significance and clarify whether this structural variation affects bacterial adhesion, biofilm formation or host immune recognition. Based on the conserved epitope regions (GAGBD and G5 domains) identified in this study, segmented expression and epitope screening assays can be performed to systematically evaluate their immunoprotective efficacy as broad-spectrum vaccine candidate antigens and explore design strategies for multivalent or multi-epitope vaccines to overcome the limitation of insufficient antigen coverage. Meanwhile, the synergistic effects of HP0197 with other virulence factors (such as HP1036, Enolase, Sao, etc.) in pathogenesis and immunity should be investigated to explore the feasibility of multi-target combined vaccines to achieve more comprehensive immune protection [23,24].

In addition, this study revealed the application potential of HP0197 beyond immune protection. Based on the highly conserved characteristic of its GAGBD that specifically binds glycosaminoglycans (GAGs) on host cell surfaces, it can be exploited as an active targeting element for infection foci. By conjugation with antibacterial agents, drugs can be prioritized to enrich in the lesion microenvironment caused by *S. suis* infection, thereby enhancing the bactericidal effect and providing a unique strategy to advance the development of novel targeted drugs against *S. suis* infection.

## 5. Conclusions

In conclusion, this study systematically analyzed the HP0197 protein family in Streptococcus suis, revealing its wide distribution and significant polymorphism classified into seven phylogroups. We identified conserved and type-specific epitopes, proposed cross-protection patterns, and highlighted the GAGBD’s potential for targeted drug delivery. These findings provide a foundation for improved vaccines and novel therapies against *S. suis* infection.

## Figures and Tables

**Table 1:** Bioinformatics analysis software used in this study.

Software	Function	Website
Protparam	Physicochemical properties	https://web.expasy.org/protparam/ (accessed on 8 January 2026)
ProtScale	Hydrophobicity	https://web.expasy.org/protscale/ (accessed on 8 January 2026)
SignalP-6.0	Signal peptide	https://services.healthtech.dtu.dk/services/SignalP-6.0/ (accessed on 8 January 2026)
TMHMM-2.0	Transmembrane structure	https://services.healthtech.dtu.dk/services/TMHMM-2.0/ (accessed on 8 January 2026)
PSORTB	Subcellular localization	https://www.psort.org/psortb/ (accessed on 8 January 2026)
SOPMA	Secondary structure	https://npsa.lyon.inserm.fr/cgi-bin/npsa_automat.pl?page=/NPSA/npsa_sopma.html (accessed on 8 January 2026)
AlphaFold3	Tertiary structure	https://alphafoldserver.com/ (accessed on 10 January 2026)

**Table 4:** Physicochemical properties of HP0197 proteins from six different phylogenetic phylogroups.

Sequence Type	Protein Length	Molecular Weight	Isoelectric Point	Hydropathy Index	Subcellular Localization
HP0197 I	561	61.3	4.85	−0.858	Bacterial cell wall
HP0197 II	528	58.2	4.81	−0.801	Bacterial cell wall
HP0197 III	519	57.2	4.92	−0.781	Bacterial cell wall
HP0197 IV	802	89.1	5.18	−0.715	Bacterial cell wall
HP0197 V	836	92.8	5.04	−0.792	Bacterial cell wall
HP0197 VI	702	77.9	5.27	−0.761	Bacterial cell wall

**Table 5:** Secondary structure prediction of HP0197 proteins from six different phylogenetic phylogroups.

Sequence Type	Alpha-Helix (%)	Beta-Sheet (%)	Random Coil (%)
HP0197 I	15.15	0	84.85
HP0197 II	14.58	1.33	84.09
HP0197 III	13.87	1.54	84.59
HP0197 IV	11.22	2.87	85.91
HP0197 V	21.51	5.70	72.79
HP0197 VI	9.69	2.15	88.16

**Table 6:** Predicted B-cell epitope sites of HP0197 in six phylogenetic phylogroups.

HP0197 I	HP0197 II	HP0197 III	HP0197 IV	HP0197 V	HP0197 VI
TTETSTATQVEAMAKV 41–56 ^1^	TEPTKTPLQHLRELVE 48–63	TTETSTATQVEAMAKV 41–56	TTETSTATQVEAMAKV 41–56	EPTVQITGTVSTENTN 60–75	TTETSTATQVEAMAKV 41–56
PSYGDAQDYSYQKALW 77–92	KYEKSAYPTQDYLWTE 77–92	TETTKTPLQHLKELVE 48–63	EPTVQITGTVSTENTN 60–75	EVPAISETPVAEKADP 87–102	EPTVQITGTVSTENTN 60–75
PGDNTIEEEYNAHLKQ 147–162	NKEIEEEYKQHLEQTE 146–161	KYLWDDNLKYGKGQMD 87–102	PEKSEEPTPAPAALTS 109–124	PEKSEEPTPAPAALTS 115–130	TGTVSTENTNSESKVT 66–81
EEEYNAHLKQDEGKSI 153–168	TEIKTYASQEGFNLKT 160–175	PAPVPEAPTPKGDAEA 201–216	RDYTGSDYSFQKGLWT 162–177	EGDAKIQSVYGRLADF 201–216	TKSAEQKVASEPEKTE 98–113
APKEETPAPKEEDTP 215–230	TNEAEQAVERIKKIVQ 177–192	KGDAEAPKDEKEVPAP 211–226	SYASQEGKVFEAYVDS 252–267	LEQREDSGTFSQEGDP 250–265	PEKTEEPNPTPAALTS 109–124
APKEEDTPAPDAAPAP 223–238	ETKEDEVPVPTPEVDP 204–219	GVPTPAPVPETPMDEP 246–261	DSAEQAFDRIVNRVNE 266–281	TFSQEGDPLRRSVEKS 258–273	KMLWADSLEFGKKHMD 173–188
APDAAPAPATPFEVDP 231–246	PAPIPDTPKAEEDAPT 219–234	EEPKTDKVESDKQMPE 309–324	TKEQEDPNLPLGEKVE 321–336	LQRIKERAEEIEGDNL 277–292	GKKHMDYVRNLKKGTP 183–198
PKAEEAPTPYPDPTA 255–270	PKAEEDAPTPVPDTPA 226–241	PKAEDMPKEEMPKAEQ 333–348	SETYQDVIVGDKIVAS 346–361	SETYQDVIVGDTIVAS 351–366	EKEYKAHLEQREDSGT 236–251
PAPIPDAPTPKVEET 277–292	KEEGIPAPMPEAPAPK 274–289	ALPIKEEIRYDASLAL 385–400	GKVIESKLLSEDRKTP 450–465	GDTIVASTLISKKRKE 360–375	LKRIKERAEEIEGDNL 270–285
PETKEETTPYPDPTA 302–317	APMPEAPAPKTEEDTP 280–295	DVIVDGKVVATNLLSE 420–435	KLLSEDRKTPVNRLIA 456–471	DTAPTAPEKPKLEFTV 392–407	SETYQDVIVGDKIVAS 344–359
APTPMETPMDKPKTD 341–356	APTPMPDTPMDQPKTD 308–323	PSVQSNPTLSHKGAPS 461–476	ASTPMPETPMDKPKTD 587–602	DTAPTAPEKPKLEFTV 486–501	LREGQKSELVKKYQDV 429–444
DKFKTDEVESDKQMPE 351–366	DQPKTDKVESDKQMPE 318–333		DKPKTDKVESDKQMPE 597–612	GELIKKYQDVIVEGNV 536–551	EKTPDAPAPKAEEDTP 475–490
PKAEDMPKEEMPKSEQ 375–390	PKAEDMPKEEMPKSEQ 342–357		PEMEQPKAEDMPKAEQ 616–631	EDEALKPMEPEADKPT 586–601	APKAEEDTPMDKPKTD 482–497
PKSEQPKSEDSAPKTA 386–401	KAEDSAPKTAVPEVAP 359–374		KAEDSAPKTAVPEVAP 633–648	EADKPTPPTPEAPKTE 596–601	DKPKTDKVESDKQMPE 492–507
KSEDSAPKTAVPEPAP 392–407	ALPIKEEIRYDASLPL 394–409		ALPIKEEIRYDASLAL 668–683	PPTPEAPKTEMSDAEQ 602–617	PKAEDMPKEEMPKAEQ 516–531
ALPIKEETRYTASLPL 427–442	MKKEEVKTTPSVQSNP 461–476		KEEVKSTPSVQSNLTL 737–752	EQPKVDKPQMEAPKTD 616–631	NAEDSAPKTAVPEVAP 533–548
DVIVDGKVATNLLSE 462–477	PSVQSNPTLSHKGAPS 470–485			EAPKTDKVESDKQMPE 626–641	ALPIKEEIRYDASLAL 568–583
MKKEEVKTTPSVQSNP 494–509				PKAEDMPKEEMPKAEQ 650–665	DVIVDGKVVSTNLLSE 603–618
PSYQSNPTLSHKGAPS 503–518				KAEDSAPKTAVPEVAP 667–682	MKKEEVKTTPSVQSNP 635–650
				ALPIKEEIRYDASLAL 702–717	TPSVQSNPTMSQKGAP 643–658
				DVIVDGKVVATNLLSE 737–752	
				MKKEEVKTTPSVQSNP 769–784	
				PSVQSNPTQSHKGAPS 778–793	

^1^ amino acid start position of the epitope in each group.

## Data Availability

The original contributions presented in this study are included in the article/Supplementary Materials. Further inquiries can be directed to the corresponding author.

## Supplementary Materials

The following supporting information can be downloaded at: https://www.mdpi.com/article/10.3390/vetsci13040376/s1, Supplementary Table S1. Distribution and Similarity Analysis of HP0197 in *S. suis*.
