# Supplementary material for: Population Genetic Architecture of the Streptococcus suis Antigen HP0197
Source: Vet Sci. 2026 Apr 13;13(4):376. doi: 10.3390/vetsci13040376 (PMC13119558; doi:10.3390/vetsci13040376)
Supplement: Supplementary file 1 [file vetsci-13-00376-s001.zip › vetsci-4231445-supplementary.pdf]

Supplementary Table S1. Distribution and Similarity Analysis of HP0197 in S.suis

Number	Host	Serotype	Place of origin	Strain name	Accession number	DNA lengh （bp）	Similary （%）	Coverage （%）	Classification
1	Swine	1/2	China	SS12	CP002640.	1545	99.20	98	Ⅰ
2	Swine	1	China	ST1	CP002651.	2433	97.46	51	Ⅳ
3	Swine	1	Canada	Ssuis_MA1	CP085088.	1686	99.94	100	Ⅰ
4	Swine	1	Thailand	ID35541	CP109942.	1896	96.76	100	Ⅰ
5	Swine	1	Thailand	TRG6	CP109937.	1560	93.30	52	Ⅲ
6	Swine	1	The Netherlands	NLS40	CP134477.	2151	97.73	51	Ⅳ
7	Swine	2	China	SC19	CP020863.	1686	100.00	100	Ⅰ
8	Swine	2	China	ZY05719	CP007497.	1686	100.00	100	Ⅰ
9	Swine	2	China	SS2-1	CP018908.	1686	100.00	100	Ⅰ
10	Swine	2	China	CS100322	CP024050.	1686	99.94	100	Ⅰ
11	Swine	2	China	SC070731	CP003922.	1686	99.94	100	Ⅰ
12	Swine	2	China	98HAH33	CP000408.	1686	99.94	100	Ⅰ
13	Swine	2	China	A7	CP002570.	1686	99.94	100	Ⅰ
14	Swine	2	China	TJS56	CP095463.	1686	99.94	100	Ⅰ
15	Swine	2	China	SC183	CP071305.	2061	96.51	54	Ⅲ
16	Swine	2	China	TJS75	CP095162.	2409	96.05	51	Ⅳ
17	Swine	2	China	HA0609	CP024126.	2409	95.91	51	Ⅳ
18	Swine	2	China	05HAS68	CP002007.	2409	95.91	51	Ⅳ
19	Swine	2	The United States	ISU2714	CP030022.	1686	99.94	100	Ⅰ
20	Swine	2	The United States	ISU1606	CP030017.	1686	99.94	100	Ⅰ
21	Swine	2	The United States	ISU2514	CP030020.	2409	96.18	51	Ⅳ
22	Swine	2	The United States	ISU2660	CP031379.	2409	95.91	51	Ⅳ
23	Swine	2	The United States	ISU2414	CP030023.	2409	95.91	51	Ⅳ
24	Swine	2	The United States	ISU2614	CP031377.	2409	95.91	51	Ⅳ
25	Swine	2	Spain	SS15055_N2_C	CP102154.	1686	99.94	100	Ⅰ
26	Swine	2	Spain	M104300_S20	CP102137.	1686	99.94	100	Ⅰ
27	Swine	2	Europe	P1/7	AM94601	1686	99.94	100	Ⅰ
28	Swine	2	Germany	DE609B	CP100435.	1686	99.88	100	Ⅰ
29	Swine	2	The Netherlands	10	CP058742.	1686	99.94	100	Ⅰ
30	Swine	2	The Netherlands	S10	LR738721.	1686	99.94	100	Ⅰ
31	Swine	2	The Netherlands	S735	CP003736.	1899	96.85	100	Ⅰ
32	Swine	2	The Netherlands	GD-0001	LR738720.	1899	96.85	100	Ⅰ
33	Swine	2	Canada	NSUI060	CP012911.	2409	96.18	51	Ⅳ
34	Swine	2	Canada	90-1330	CP012731.	2409	96.03	51	Ⅳ
35	Swine	2	Canada	NSUI002	CP011419.	2409	95.91	51	Ⅳ
36	Swine	2	Denmark	DNR43	CP102143.	2409	95.91	51	Ⅳ
37	Swine	2	Denmark	DNC49	CP102140.	2409	95.91	51	Ⅳ
38	Swine	3	China	YA	CP149804.	2124	96.33	51	Ⅳ
39	Swine	3	China	YB51	CP006645.	2409	95.91	51	Ⅳ
40	Swine	3	China	ST3	CP002633.	1215	95.91	51	Ⅳ
41	Swine	3	Germany	PH2016-081	CP134474.	1608	98.70	99	Ⅰ
42	Swine	3	South Korea	INT-01	CP041994.	2409	96.03	51	Ⅳ
43	Swine	3	Canada	Ssuis_MA8	CP085085.	2409	95.91	51	Ⅳ
44	Swine	3	Canada	MY1C3_3B	CP134487.	1560	93.30	52	Ⅲ
45	Swine	4	China	SH1510	CP030124.	1560	93.30	52	Ⅲ
46	Swine	4	China	HA1003	CP030125.	2511	96.88	47	Ⅴ
47	Swine	4	Denmark	6407	CP008921.	2373	99.62	50	Ⅵ
48	Swine	4	The Netherlands	Ssuis_MA6	CP085086.	1803	96.40	100	Ⅰ
49	Swine	4	Canada	Ssuis_MA2	CP085087.	1803	96.40	100	Ⅰ
50	Swine	4	Thailand	3453	CP199993.	1560	93.30	47	Ⅲ
51	Swine	4	Thailand	ID34572	CP109940.	1560	93.30	52	Ⅲ

52	Swine	5	China	HN105	CP029398.	2511	97.03	47	Ⅴ
53	Swine	5	China	SH0104	CP025419.	2409	95.91	51	Ⅳ
54	Swine	5	Thailand	ID26102	CP135087.	2769	96.90	56	Ⅴ
55	Swine	5	Thailand	ID34567	CP135089.	2511	95.94	47	Ⅴ
56	Swine	5	Thailand	ID32563	CP135063.	2511	95.94	47	Ⅴ
57	Swine	7	China	D9	CP002641.	888	96.05	51	Ⅳ
58	Swine	7	Germany	13-00283-02	CP058741.	2409	96.18	51	Ⅳ
59	Swine	8	China	2018WUSS151	CP101844.	2073	96.03	51	Ⅳ
60	Swine	8	China	WUSS030	CP110141.	2073	94.48	51	Ⅳ
61	Swine	8	Denmark	DNR48	CP102141.	2373	99.62	50	Ⅳ
62	Swine	9	China	D12	CP002644.	2733	97.04	46	Ⅲ
63	Swine	9	China	NJ3	CP082203.	2511	97.03	47	Ⅴ
64	Swine	9	China	DN13	CP015557.	1848	89.09	53	Ⅲ
65	Swine	9	China	GZ0565	CP017142.	1848	89.09	53	Ⅲ
66	Swine	9	Germany	16085/3b	CP058740.	1539	97.15	46	Ⅲ
67	Swine	9	The Netherlands	NLS50	CP134488.	1806	96.40	100	Ⅰ
68	Swine	9	The Netherlands	9401240	LR738724.	2511	96.72	47	Ⅴ
69	Swine	9	Denmark	DNS11	CP102152.	1899	96.21	100	Ⅰ
70	Swine	9	Spain	M102942_S11	CP102138.	1560	93.30	52	Ⅲ
71	Swine	12	The United States	ISU2912	CP017785.	2631	94.72	60	Ⅱ
72	Swine	14	China	JS14	CP002465.	1686	99.94	100	Ⅰ
73	Swine	16	China	TL13	CP003993.	1476	91.36	59	Ⅱ
74	Swine	16	The United Kingdom	LSS42	CP100430.	2439	99.15	51	Ⅴ
75	Swine	16	Denmark	DNC15	CP102148.	1848	97.34	46	Ⅲ
76	Swine	19	Spain	M105052_S26	CP102136.	2109	96.92	48	Ⅴ
77	Swine	19	Canada	89-4294	CP134478.	2109	96.76	48	Ⅴ
78	Swine	19	The United States	ISU2812	CP030015.	2109	96.76	48	Ⅴ
79	Swine	21	Spain	M104170_C2	CP134469.	1890	97.19	57	Ⅲ
80	Swine	23	Canada	1522228	CP134472.	1560	93.16	52	Ⅲ
81	Swine	29	The United Kingdom	TMW_SS028	CP134473.	2109	96.92	48	Ⅴ
82	Swine	30	Spain	M106471_S40	CP102135.	2520	97.70	45	Ⅲ
83	Swine	31	China	1081	CP017667.	2511	97.03	47	Ⅴ
84	Swine	31	China	0061	CP017666.	2511	97.03	47	Ⅴ
85	Swine	31	China	SS389	CP082202.	2511	97.03	47	Ⅴ
86	Swine	31	Thailand	35539	CP185356.	2511	97.62	47	Ⅴ
87	Swine	31	Thailand	35540	CP185358.	2511	97.62	47	Ⅴ
88	Swine	31	Thailand	35542	CP185360.	2511	97.62	47	Ⅴ
89	Swine	31	Thailand	35543	CP185362.	2511	97.62	47	Ⅴ
90	Swine	31	Thailand	35545	CP185366.	2511	97.62	47	Ⅴ
91	Swine	31	Thailand	34410	CP185347.	2511	97.49	47	Ⅴ
92	Swine	31	Thailand	34562	CP185350.	2511	97.31	47	Ⅴ
93	Swine	31	Thailand	34558	CP185349.	2511	97.19	47	Ⅴ
94	Swine	31	Thailand	34684	CP185352.	2511	97.19	47	Ⅴ
95	Swine	31	Thailand	34692	CP185353.	2511	97.19	47	Ⅴ
96	Swine	31	Thailand	35538	CP185354.	2511	97.19	47	Ⅴ
97	Swine	31	Thailand	35544	CP185364.	2511	97.19	47	Ⅴ
98	Swine	31	Thailand	STC501	CP185368.	2511	97.15	47	Ⅴ
99	Swine	31	Thailand	STC516	CP185369.	2511	97.15	47	Ⅴ
100	Swine	31	Thailand	34550	CP185348.	2511	96.99	47	Ⅴ
101	Swine	31	Thailand	34566	CP185351.	2511	96.56	47	Ⅴ
102	Swine	Chz	China	HN136	CP025095.	2556	97.29	76	Ⅵ
103	Swine	Chz	China	AH681	CP025043.	2505	97.27	91	Ⅰ
104	Swine	Chz	China	CZ130302	CP024974.	1821	91.72	90	Ⅰ
105	Swine	NCL1	China	YSJ17	CP032064.	3162	97.01	60	Ⅱ
106	Swine	NCL3	China	AKJ18	CP082205.	1968	97.41	62	Ⅱ

107	Swine	NCL3	China	YZDH1	CP065430.	2109	96.92	48	Ⅴ
108	Swine	NCL3	China	2022WUSS148	CP140109.	2109	96.92	48	Ⅴ
109	Swine	-[1]	China	HB18	CP141824.	1899	96.48	100	Ⅰ
110	Swine	-	China	SS2404	CP199721.	2457	98.17	55	Ⅳ
111	Swine	-	China	SS2422	CP199400.	2409	95.91	51	Ⅳ
112	Swine	-	China	HN38	CP116604.	1821	91.72	77	Ⅵ
113	Swine	-	China	1112S	CP071697.	3354	91.60	47	Ⅲ
114	Swine	-	The United States	SRD478	CP030010.	2388	96.93	40	Ⅴ
115	Swine	-	Canada	1521251	CP100431.	1560	93.30	52	Ⅲ
116	Subjects	1	Thailand	ID38828	CP109941.	1638	99.70	100	Ⅰ
117	Subjects	2	China	SC84	FM252031	1686	100.00	100	Ⅰ
118	Subjects	2	China	05ZYH33	CP000407.	1686	100.00	100	Ⅰ
119	Subjects	2	China	LSM178	CP047248.	1686	99.94	100	Ⅰ
120	Subjects	2	China	SZ1908	CP082948.	1686	99.94	100	Ⅰ
121	Subjects	2	China	LSM102	CP016175.	1686	99.94	100	Ⅰ
122	Subjects	2	China	GZ1	CP000837.	2304	97.15	43	Ⅶ
123	Subjects	2	Vietnam	BM407	FM252032	1890	99.60	100	Ⅰ
124	Subjects	2	The Netherlands	861160	LR738722.	1899	96.85	100	Ⅰ
125	Subjects	2	Thailand	STC84	CP100332.	1587	91.84	67	Ⅱ
126	Subjects	2	Thailand	STC104	CP100418.	1587	91.84	67	Ⅱ
127	Subjects	2	Thailand	STC90	CP100326.	1587	91.84	67	Ⅱ
128	Subjects	2	Thailand	STC86	CP100328.	1587	91.84	67	Ⅱ
129	Subjects	2	Thailand	STC85	CP100330.	1587	91.84	67	Ⅱ
130	Subjects	2	Thailand	STC83	CP100334.	1587	91.84	67	Ⅱ
131	Subjects	2	Thailand	STC81	CP100336.	1587	91.84	67	Ⅱ
132	Subjects	2	Thailand	STC80	CP100338.	1587	91.84	67	Ⅱ
133	Subjects	2	Thailand	STC78	CP100340.	1587	91.84	67	Ⅱ
134	Subjects	3	China	Ss2301	CP152119.	1686	99.94	100	Ⅰ
135	Subjects	4	Thailand	ID36054	CP109939.	1560	93.30	52	Ⅲ
136	Subjects	5	Thailand	ID48908	CP135090.	2769	96.90	56	Ⅴ
137	Subjects	5	Thailand	ID41570	CP079193.	2511	96.88	47	Ⅴ
138	Subjects	5	Thailand	ID24665	CP135065.	2511	96.35	47	Ⅴ
139	Subjects	5	The United States	1547095	CP135094.	1560	93.30	52	Ⅲ
140	Subjects	5	Argentina	1652329	CP135093.	1560	93.30	52	Ⅲ
141	Subjects	7	China	GX69	CP071806.	1560	93.30	52	Ⅲ
142	Subjects	24	Thailand	ID32098	CP082778.	2511	96.88	47	Ⅴ
143	Subjects	24	Thailand	ID33329	CP068708.	2511	96.88	47	Ⅴ
144	Subjects	24	Thailand	39565	CP076517.	2511	96.88	47	Ⅴ
145	Subjects	-	China	HBXY-SL	CP121670.	1686	99.94	100	Ⅰ
146	Subjects	-	China	LSM29	CP091423.	1686	99.94	100	Ⅰ
147	Subjects	-	China	LSM157	CP091422.	1686	99.94	100	Ⅰ
148	Subjects	-	China	SC2022MYS16	CP195773.	2124	96.33	51	Ⅳ
149	Giant gourami	6	Thailand	3112	CP097577.	2082	94.65	87	Ⅵ
150	Cattle	-	Canada	KKAHC02	CP176418.	2511	97.03	47	Ⅴ
151	-	2	Japan	DAT299	AP023391.	2421	98.87	51	Ⅳ
152	-	2	The United Kingdom	NCTC10234	LS483418.	1899	96.85	100	Ⅰ
153	-	15	The Netherlands	T15	CP006246.	1608	98.70	99	Ⅰ
154	-	S	-	NCTC10237	LR594043.	2433	97.46	51	Ⅳ
155	-	-	Japan	DAT300	AP023392.1	1848	89.09	53	Ⅲ

[1]No host or serotype information was found.
